# Comparative analysis of exosome isolation methods using culture supernatant for optimum yield, purity and downstream applications

**DOI:** 10.1038/s41598-019-41800-2

**Published:** 2019-03-29

**Authors:** Girijesh Kumar Patel, Mohammad Aslam Khan, Haseeb Zubair, Sanjeev Kumar Srivastava, Moh’d Khushman, Seema Singh, Ajay Pratap Singh

**Affiliations:** 1 Department of Oncologic Sciences, Mitchell Cancer Institute, University of South Alabama, Mobile, Alabama USA; 2 Department of Medical Oncology, Mitchell Cancer Institute, University of South Alabama, Mobile, Alabama USA; 3 Department of Biochemistry and Molecular Biology, College of Medicine, University of South Alabama, Mobile, Alabama USA

## Abstract

Exosomes have received significant attention for their role in pathobiological processes and are being explored as a tool for disease diagnosis and management. Consequently, various isolation methods based on different principles have been developed for exosome isolation. Here we compared the efficacy of four kits from Invitrogen, 101Bio, Wako and iZON along with conventional ultracentrifugation-based method for exosome yield, purity and quality. Cell culture supernatant was used as an abundant source of exosomes, and exosome quantity, size-distribution, zeta-potential, marker-expression and RNA/protein quality were determined. The Invitrogen kit gave the highest yield but the preparation showed broader size-distribution likely due to microvesicle co-precipitation and had the least dispersion stability. Other preparations showed <150 nm size range and good stability. Preparation from iZON column; however, had a broader size-distribution in the lower size range suggestive of some impurities of non-vesicular aggregates. RNA quality from all preparations was comparable; however, proteins from Invitrogen method-based exosomal preparation showed polyethylene glycol (PEG) contamination in mass spectrometry. Chemical impurities from the precipitant could also be the cause of toxicity of Invitrogen method-based exosomal preparation in biological growth measurement assay. Together, these findings should serve as a guide to choose and further optimize exosome isolation methods for their desired downstream applications.

## Introduction

Exosomes are small membrane vesicles (30–150 nm) of endocytic origin, which are shed by all cell types under normal- and patho-physiological conditions. They are released to extracellular milieu in exocytic bursts upon fusion of the multiple vesicular bodies (MVBs) to the cell membrane and are found in abundance in body fluids including blood, urine, saliva, milk, semen, bile juice, ascites, cystic, bronchoalveolar and gastrointestinal lavage fluid^1–3^. Exosome contains variety of pivotal molecules such as proteins, nucleic acids (microRNA, long-noncoding RNA, intact and mutated mRNA and fragments of DNA), lipids and some other metabolites (amino acids, sugars, etc.)^2,4,5^, and their composition are affected by different environmental factors and health status^6–8^.

Exosomes play an important role in inter-cellular communication and are capable of modulating the recipient cell behavior by autocrine^9^, paracrine^10,11^, endocrine and/or juxtracrine^12^ modes of cell signaling. They have received considerable attention lately due to unveiling of their various novel roles in cancer progression, angiogenesis, metastatic niche formation, organ-specific metastasis, tumor microenvironment remodeling, immune suppression, etc.^2,13–15^. In addition, exosomes in patient’s body-fluids have emerged as a promising source for biomarker development^5^. They can be isolated from the small amount of biological fluids and clinical samples and their cargo, which represents tissue-specific molecules with higher stability, can serve as disease-specific biomarkers^16,17^. Furthermore, since their release and composition can be modulated by environmental factors, they can also serve as markers for disease status and treatment outcomes^18,19^. Moreover, being natural carrier of biomolecules, effort have been made to potentially exploit exosomes as a stable and targeted drug delivery system^16,20,21^.

With increasing potential for their clinical utilization, it has become imperative to optimize their isolation method for maximum yield, purity and assay reproducibility. Besides classical ultracentrifugation method there are currently several commercial exosome isolation kits developed based on different principles such as charge neutralization-based precipitation, gel-filtration, affinity purification using magnetic beads, etc., are available in the market. Here we have compared these methods of exosome isolation using cell culture supernatant as an abundant sample source. Our data show similarities and differences in yield, purity and integrity of isolated exosomes and demonstrate qualitative differences of exosomal preparations for downstream applications (RNA and protein analysis and functional studies). Thus, presented data from this study will serve as a guide to choose and further optimize exosome isolation methods for their desired applications in biomarker development and/or biological assays.

## Results

### Different exosome isolation methods yield different amount of exosomes

**Figure 1:**
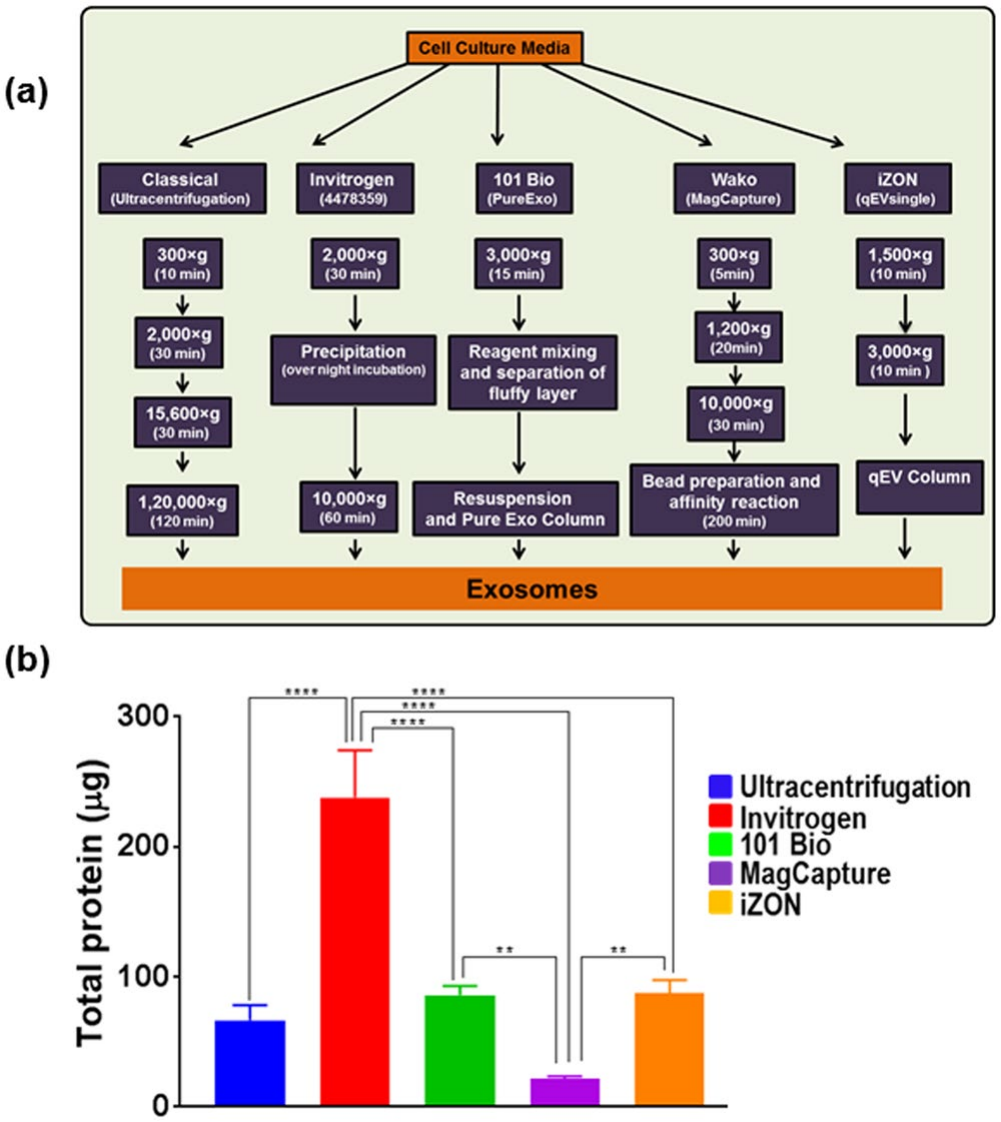
Comparison of various methodologies for exosome isolation. (**a**) Schematic representation of exosomes isolation methods. MiaPaCa cells (3 × 10^6^) were cultured in regular media, after 24 h, media was replaced with 5% exosome depleted FBS. After 48 h condition media was collected, centrifuged at 300 × g for 10 min to remove cells and cell-debris. Thereafter, exosomes were isolated using ultracentrifugation and four commercial kits following the manufacturer’s instructions. (**b**) Levels of proteins were estimated in intact exosomes by DC protein assay. Highest exosome yield was detected for Invitrogen Kit, while MagCapture yielded the least amount of exosomes. Data presented as mean ± s.d.; n = 3, p-values **<0.0093, and ****<0.0001.

To compare the isolation efficiency of different exosome isolation methods, we used culture supernatant from a pancreatic cancer line (MiaPaCa) which was available to us in abundant quantity. Exosomes were isolated as per the manufacturers’ recommended instruction for commercial kits, whereas differential speed centrifugation was done for classical ultracentrifugation method (Fig. 1a**)**. Total exosome yield was determined by protein estimation from intact exosomes using the protein DC assay kit. We observed that the precipitation-based Total Exosomes Isolation kit (Invitrogen) had the maximum yield followed by gel-filtration chromatography (iZON, qEVSingle), PureExo kit (101Bio), and differential ultracentrifugation. However, the affinity-based MagCapure exosome isolation method yielded the least amount of exosomes (Fig. 1b).

### Size distribution of exosomes significantly varies among preparations from different isolation methods

**Figure 2:**
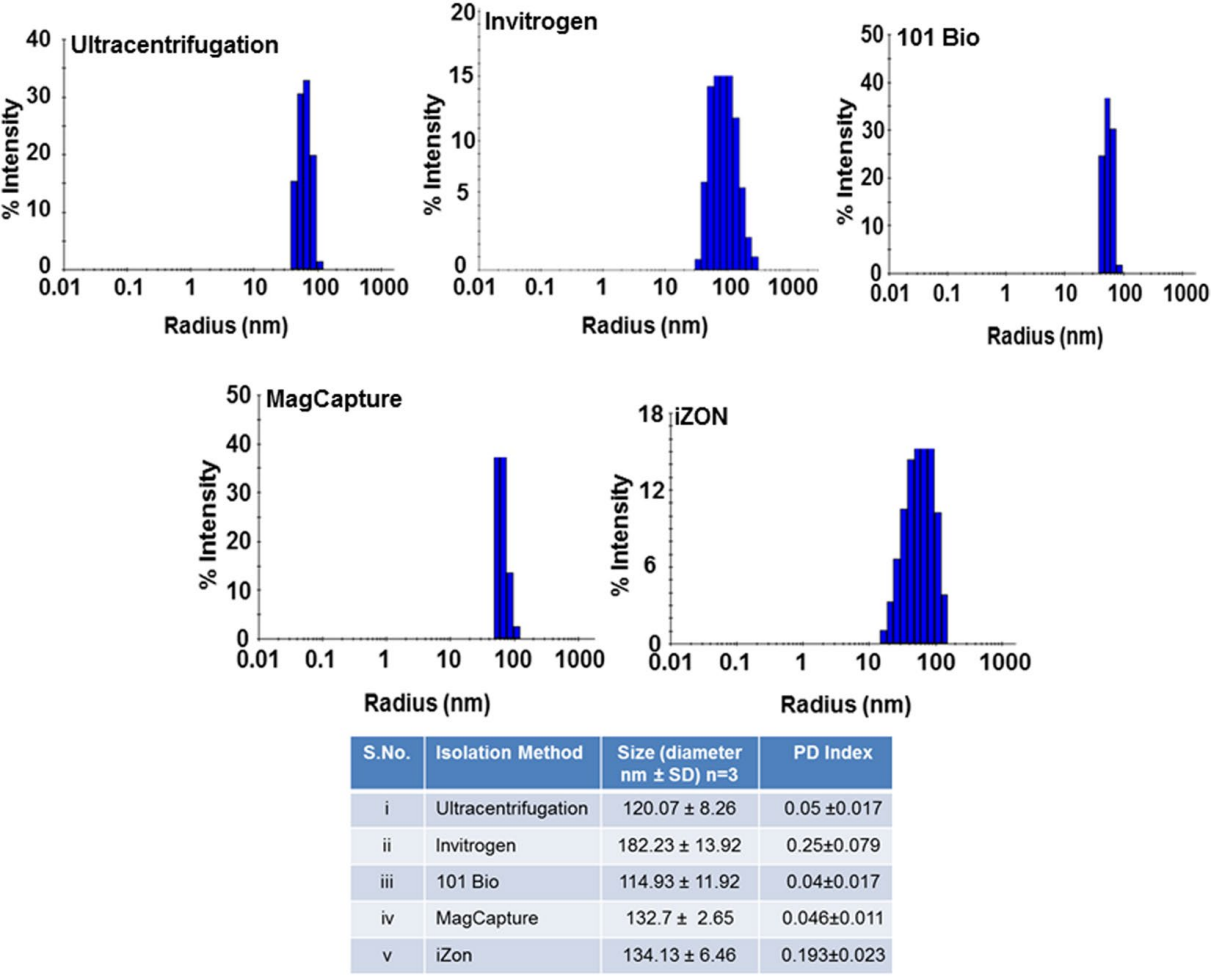
Size distribution and Polydispersity index (PDI) analysis of using dynamic light scattering. To determine the size distribution, freshly isolated exosomes were subjected to dynamic light scattering measurements using DelsaMax Pro (Backman Coulter Inc.). Exosomes isolated using Ultracentrifugation, 101Bio (PureExo), MagCapture and iZON gel-filtration, were under the size range (<150 nm), while the exosomes isolated from Invitrogen precipitation method showed broader size distribution with a shift towards the bigger size (182 ± 13.93). Data presented as mean ± s.d.; n = 3.

To evaluate the size distribution, freshly isolated exosomes from different methods were subjected to dynamic light scattering measurements, DelsaMax Pro (Beckman Coulter, Indianapolis, IN, USA). All the exosome preparations from different isolation methods showed the accepted size range (<150 nm) except those resulting from Invitrogen exosomes isolation method (Fig. 2). The latter showed a broad size distribution with a shift towards overall average bigger size (182 ± 13.92 nm). Furthermore, PD index of exosome prep from Invitrogen method was higher (0.25 ± 0.079) as compared to that of other preps (below 0.05 except for iZON method, which was closer to 0.19 ± 0.023) indicating that the preparation was highly heterogeneous. The 101Bio kit-based isolation yielded exosomes of overall lowest average size (114.93 ± 11.92 nm) with PD index of 0.04 ± 0.017 suggesting that this had the most homogeneous size distribution. Classical exosomes isolation (Ultracentrifugation) and affinity-based (MagCapture) methods also showed a narrow size range of homogeneous distribution 120.07 ± 8.26 nm and 132.7 ± 2.65 nm, respectively with PD index of 0.05 ± 0.017and 0.046 ± 0.011, respectively (Fig. 2).

### Exosomes isolated from different isolation methods show differences in their zeta potential

**Figure 3:**
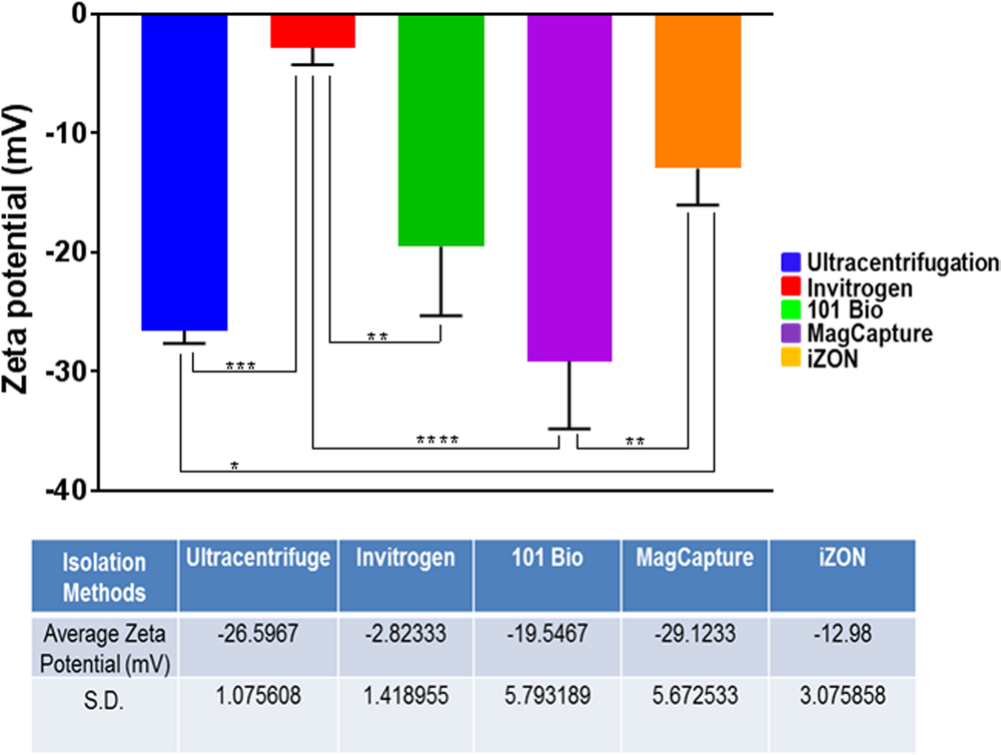
Measurement of dispersion stability. Zeta potential was estimated by Dynamic Light Scattering equipped with Phase Analysis Light Scatter (PALS). Values were calculated from measured velocities using Smoluchowski equation. Data presented as mean ± s.d.; n = 3, p-value **<0.012, ***<0.0002, and ****<0.0001.

To evaluate exosomal stability and integrity, zeta potential of freshly isolated exosomes was recorded using PALS measurements and values were calculated from measured velocities using Smoluchowski equation. A higher magnitude of the zeta potential indicates higher repulsion between the particles in suspension suggesting high dispersion stability. The exosomal preparations from MagCapture, ultracentrifugation, 101 Bio and iZON gel filtration methods showed an averaged negative surface charge and exhibited a zeta potential of −29.12 mV, −26.59 mV, −19.54 mV and −12.98 mV, respectively (Fig. 3). Surprisingly, the exosomal preparation from Invitrogen precipitation method showed the least negative surface charge (−2.82 mV).

### Marker-based assessment of exosomes isolated using different methods

**Figure 4:**
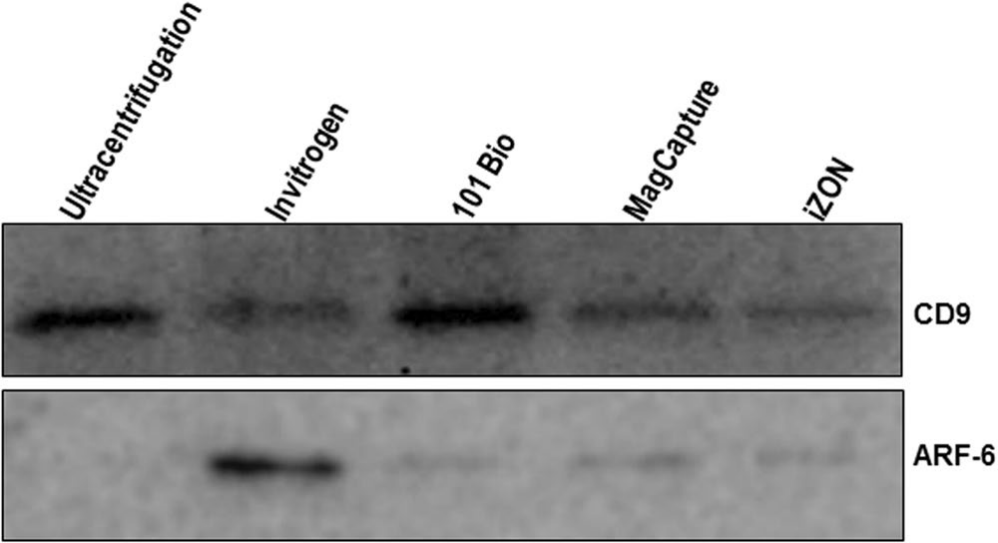
Measurement of exosome purity. A total of 25 µg protein from different exosomal preparations was resolved on 12% SDS-PAGE gel and immunoblotted using antibodies against specific marker proteins (CD9 for exosomes) and (ARF6 for microvesicles). Chemo-luminescence signal was detected under ChemiDoc gel imager (Bio-Rad) and photographed. Representative images are shown.

As we observed that the size distribution of exosomal preparations from, different isolation methods varied, we examined their purity by immunoblotting for specific marker proteins. CD9 is a specific marker for exosomes, whereas ARF6 is detected in the microvesicles^7,22,23^. We observed that all the preparations from different isolation methods showed different intensity signal for CD9 marker with least intensity detected for prep from Invitrogen method (Fig. 4; Supplementary Figure). In contrast, we detected only faint signal for ARF-6 in all preparation except that from Invitrogen precipitation method suggesting that the latter had the presence of microvesicles (Fig. 4).

### No major differences in RNA quantity and quality are observed in exosomal preparations from different methods

**Figure 5:**
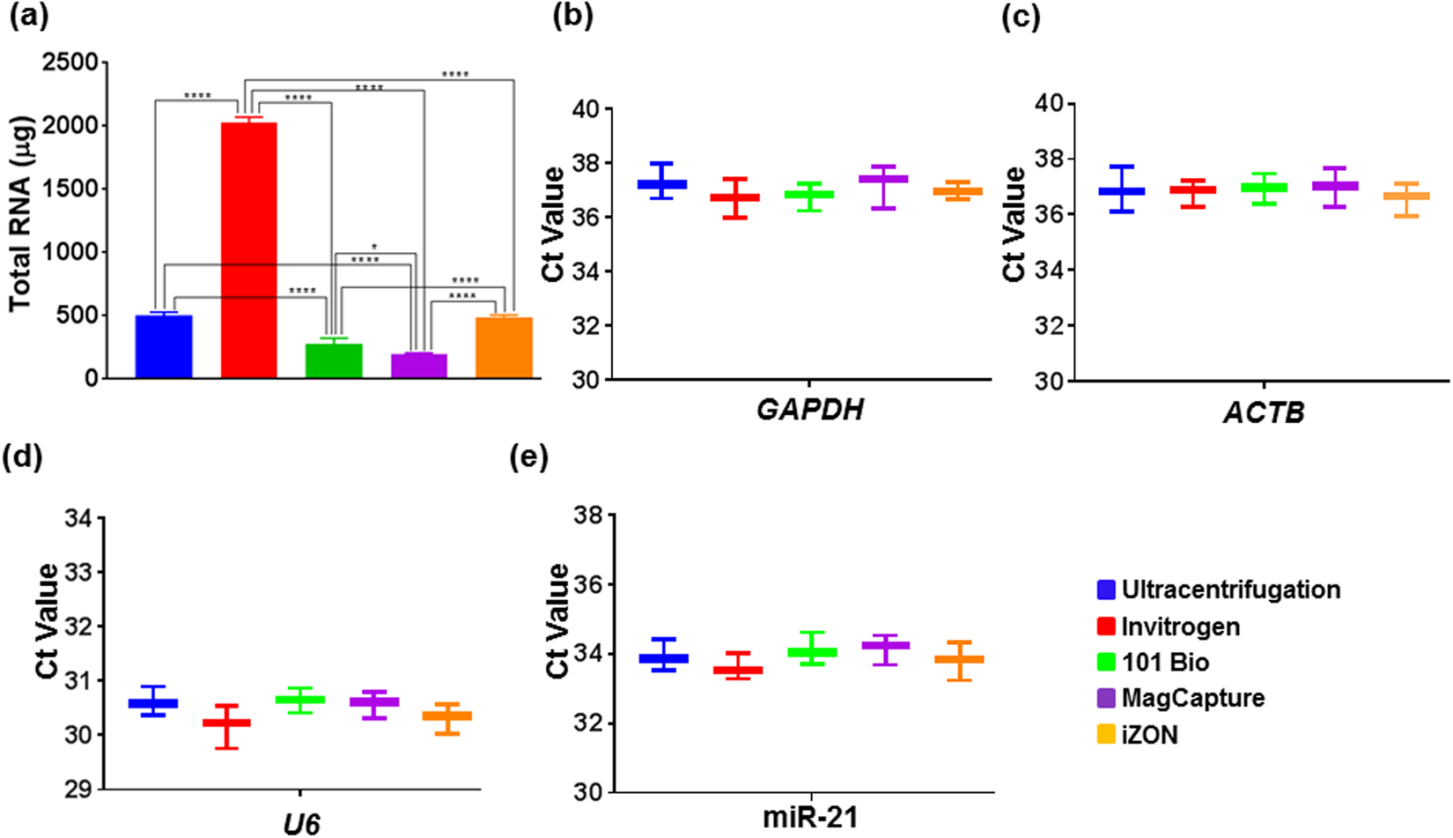
RNA quantification and quantitative real-time PCR. (**a**) RNA was isolated from different exosomal preps using total exosome RNA isolation kit and quantified using nanodrop-1000. Differential RNA yield reflected different exosome quantity obtained from various procedures. (**b**) cDNA was prepared using 40 ng of RNA using specific RT primer for microRNA while Random primer were used for GAPDH and β-actin and expression of U6, miR-21, β-actin and GAPDH was examined by qRT-PCR. Ct values are plotted for each transcripts and data presented as mean ± s.d.; n = 3, p-values *≤0.0246, and ****<0.0001.

Total RNA was isolated from the total amount of exosomes yielded by different methods and quantified using Nanodrop-1000 (Thermo Fisher Scientific, Wilmington, DE, USA). The RNA quantity from different preparations was reflective of the total exosomes yielded by isolation methods (Fig. 5a). As Invitrogen’s method provided the most amounts of exosomes it also provided the maximum yield of total RNA, while the least amount of RNA was obtained from MagCapture exosome preparation (Fig. 5a). To assess the quality of RNA for downstream application, we used equal amount of RNA (40 ng) from each preparation and used for the cDNA synthesis. Subsequently, PCR amplification of different transcripts (*GAPDH, ACTB*, U6 and MIR-21) was performed using specific primer sets (Table 1). No significant differences in the Ct values were detected for RNAs from different exosomal preparations suggesting that they are equally good qualitatively (Fig. 5b–e**)**.

**Table 1:** Primer’s sequence for U6, miR-21, *GAPDH* and *ACTB*.

Primer name	Sequences
U6 RT	5′-AAAATATGGAACGCTTCACGAATTTG-3′.
U6 forward	5′-CTCGCTTCGGCAGCACATATACT-3′
U6 reverse	5′-ACGCTTCACGAATTTGCGTGTC-3
miR-21 RT	5′-GTCGTATCCAGTGCAGGGTCCGAGGTATTCGCACTGGATACGACTCAACA-3′
miR-21 forward	5′-TCGGCGTAGCTTATCAGACTGA-3
Universal reverse	5′-GTCGTATCCAGTGCAGGGTCCGAGGT-3′
GAPDH forward	5′-ACAACTTTGGTATCGTGGAAGG-3′
GAPDH reverse	5′-GCCATCACGCCACAGTTTC-3′
ACTB forward	5′-CTCACCATGGATGATGATATCGC-3′
ACTB reverse	5′-AGGAATCCTTCTGACCCATGC-3′

### Mass Spectrometry analysis show differences in extracted protein quality from different exosomal preparations

**Figure 6:**
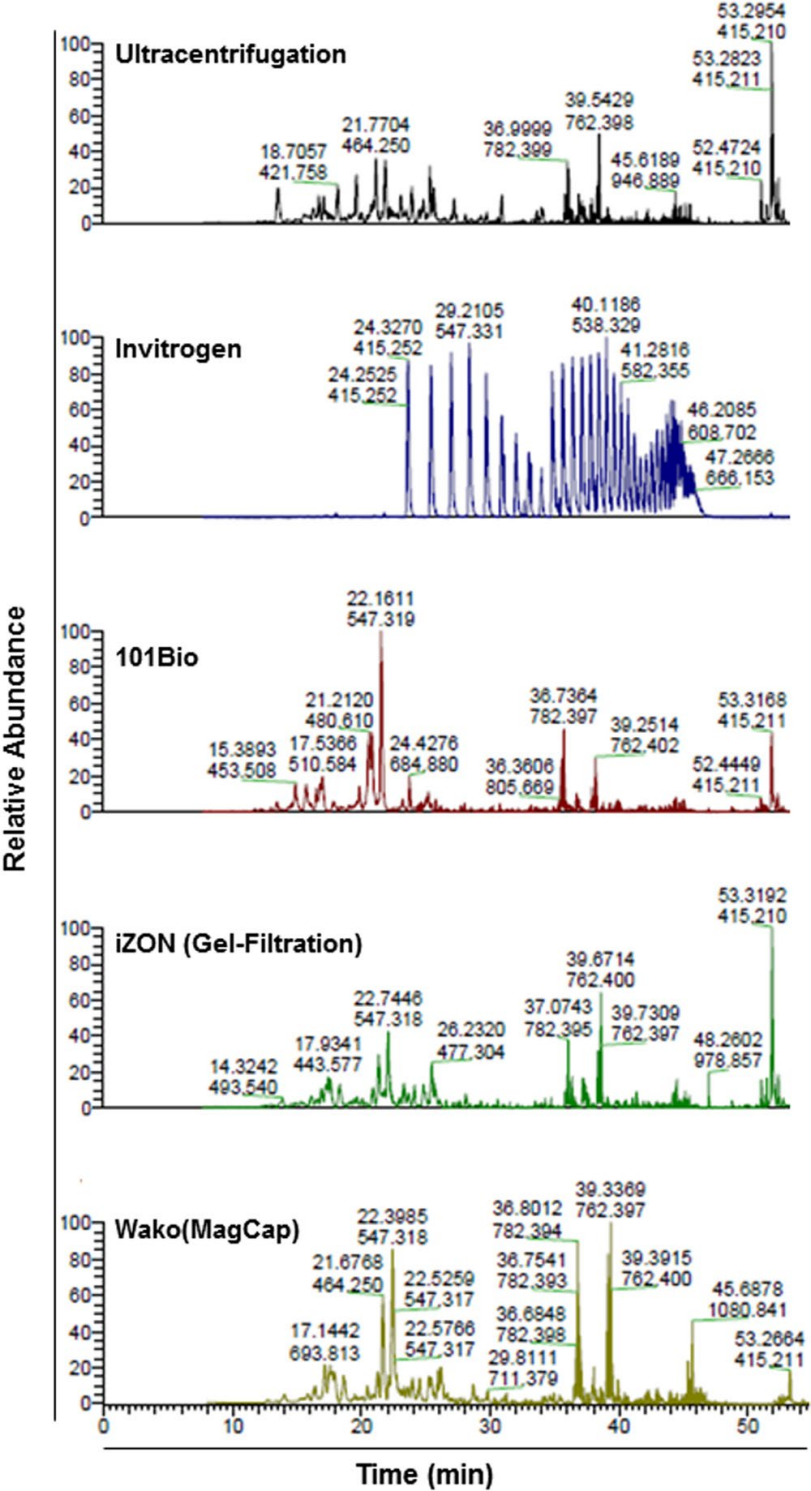
Mass spectrometry analysis of the exosomal proteins. To evaluate the compatibility of exosomes preps for proteomics study, the isolated exosome samples were dried using speed-vac and suspended into 20 µl of 8 M urea to solubilize membrane proteins followed by sonication. Subsequently, samples were incubated at −80 °C for 30 min and diluted four times by adding 60 µl of 50 mM Ammonium biocarbonate (ABC) and 10 mM Tris(2-carboxyethyl)phosphine (TCEP). Overnight digestion was done at 37 °C with sequencing grade trypsin. Samples were subjected to 1 h MS analysis on Thermo QExactive Plus mass spectrometer.

Various chemicals contaminations hinder the mass spectroscopic study of the proteins including SDS, high salt concentration, polyethylene glycols, fetal bovine serum proteins etc. So, to identify the appropriate method for exosome isolation for protein-related applications, we performed qualitative assessment using mass spectroscopic analysis. Total protein was extracted from different exosomal preparations using 8 M urea and sonication. Subsequently, proteins from each preparation were processed for mass spectrometry. The data show comparable peaks in mass spectrometry analysis of proteins from different exosomal preparations except for the one isolated using Invitrogen precipitation method (Fig. 6). Protein from Invitrogen exosomal preparation showed the presence of PEG contamination as well as the abundance of serum proteins. The protein from exosomes isolated using iZON gel filtration method also showed serum protein contamination, whereas those from ultracentrifugation, 101Bio, and MagCapture methods were of good quality for proteomic analyses (Fig. 6**)**.

### Quality of exosomes from different isolation methods for functional applications

**Figure 7:**
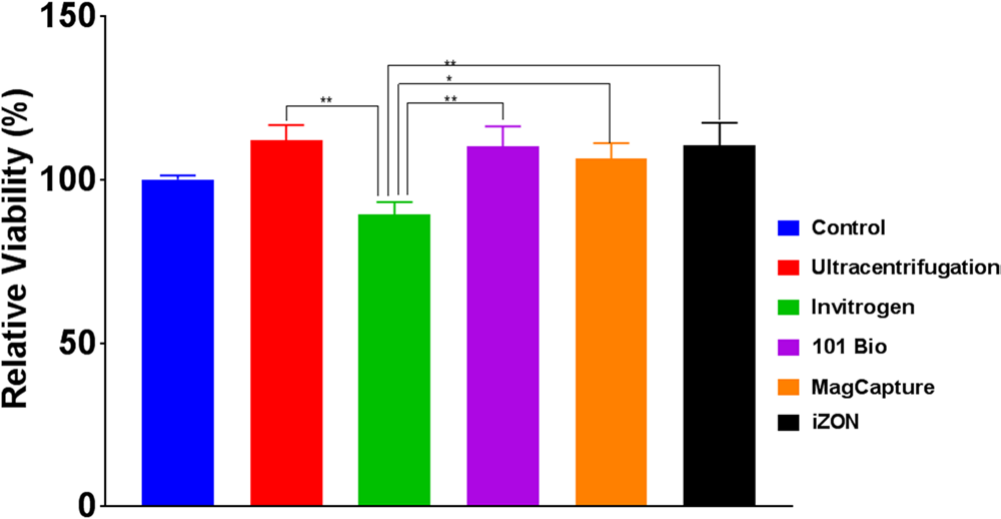
Biological effect of isolated exosomes on cells. To assess the quality of exosomal preps for functional analyses, we used isolated exosomes (10 µg/ml) for the treatment of MiaPaCa cells (5 × 10^3^) grown in 96-well plate. After 72 h, the cell proliferation assay was performed using WST-1 reagent along with control without exosome treatment. As expected we observed some growth promoting effect on MiaPaCa cells of all exosomal preps except the one isolated using Invitrogen method, which exhibited some toxicity. Data presented as mean ± s.d.; n = 3, p-value *≤0.0117, and **≤0.0065.

Since exosomes play important roles in biological processes, their isolation is being used to explore the same in functional assays. Therefore, to compare the quality of exosomal preparations, we used freshly isolated exosomes (10 μg/ml) for the treatment of pre-cultured MiaPaCa cells in 96-well plate. Following 72 h of incubation, cell proliferation was measured using WST-1 reagent. We observed that all exosomes except the ones isolated from Invitrogen method had a positive effect on cell proliferation relative to control-treated cells. This indicates that the exosomes from Invitrogen precipitation method likely contain some cytotoxic chemical(s), which inhibits the cell growth (Fig. 7).

## Discussion

Exosomes are being widely appreciated for their role in cell-cell communication and as important mediators in multiple biological functions in carcinogenesis, immunosuppression, therapy resistance, etc.^7,13,14^. Being a functionally involved entity, they are also being seen of tremendous significance in clinical and translational applications. As a result, efficient isolation of exosomes has been an active area of research to understand their biological properties and to explore their potential in biomarker development for early disease diagnosis^5,19,24^. In addition, being a natural nanoparticle with low immunogenicity, higher stability and specificity, they are also being eyed for clinical applications in drug delivery^16,20,25^. Considering this in mind, we evaluated various exosome isolation methods and generated significant data on their relative efficacy in exosome yield, purity and quality. The principle and different pros and cons of exosomes isolation methods are summarized in Table 2. Among all the methods tested, precipitation-based method of Invitrogen yielded most quantity, whereas it was the least in case of MagCapture method. All other methods yielded comparable quantity of exosomes. A likely reason for the low yield with MagCapture could be multiple washing steps that might have caused losses in exosome quantity. On the other hand, highest yield in precipitation method could be due to likely precipitation of extracellular vesicles other than just exosomes or additional aggregated proteins. It is also possible that soluble proteins from culture media also got precipitated^26^ and gave false determination of exosomes as our quantitative assay is based on protein estimation. This has also been suggested by others, who reported similar problem with the precipitation-based exosomes isolation^27,28^.

**Table 2:** Summery of different exosomes isolation methods.

Methods	Classical (Ultracentrifugation)	Invitrogen (Precipitation)	101 Bio (PureExo)	MagCapture (Affinity Based)	iZON (Size exclusion)
**Principle**	Based on density and size under centrifugal force	Differential solubility based precipitation	By precipitation and targeted filtration for removal of protein contamination	Affinity method using magnetic beads and phosphatidylserine (PS)-binding protein TIM4	Based on size exclusion chromatography
**Pros**	Good for exosomes treatment-based study, mass spectrometry analysis, RNA-seq study	Quick, easy and needs small sample size, high yield, good for small volume, RNA-seq study	Quick, easy, yield pure exosomes good for mass spectroscopy RNA-seq study	Single step, easy, no hoarse chemical, good for mass spectroscopy RNA-seq study	Fast, easy, single kit can be used for all type of sample, little volume is required
**Cons**	Time consuming, aggregated proteins and nucleic acid may be pelleted, not good for small volumes	Precipitates non- EV material, not good for mass spectrometry	Good for small volume only	Low yield, good for small volume only	Low concentrated prep, additional method for enrichment required
**Time Required**	3–4 h	12–16 h	1.5–2.0 h	4–5 h	1.5–2.0 h

In our size distribution measurements, we observed narrow size range for exosomes isolated by ultracentrifugation, 101Bio kit and MagCapture affinity-based method. Although both Invitrogen (precipitation-based) and iZON column (size-based) method yielded a relatively heterogeneous population of extracellular vesicles, average size for the latter was similar to other methods. On the other hand, average size for Invitrogen method-based preparation was relatively high with some vesicles reported to be in the range of 180–500 nm. This further supports that the Invitrogen method likely precipitates all the vesicles in the culture supernatant that were not excluded during sample pre-processing centrifugation at 2000X g for 30 min. Conversely, iZON column (size-based method) might have co-purified the protein aggregates, vitamins and other impurities from culture media during exosomal preparation^29^. Zeta potential is an indispensable factor for determining exosomal integrity and dispersion stability of exosomes^29^. We found variation (−2.8 mV to −29.12 mV) in the zeta potential of exosome preparations by different methods. Exosomes isolated by all the methods displayed negative magnitude of zeta potential similar to previous reports^30,31^. However, we observed the least negative value of exosomes isolated from Invitrogen precipitation method, which might be due to the presence of charged molecules that increased the ionic strength of the suspension medium (water). The least negative surface charge may lead to the low dispersion stability of the exosomes and may compromise the biological functions^29^.

Further, we found that as expected CD9 (exosome marker) was expressed in all exosomal preparations. However, we observed high ARF6 (microvesicle marker) expression in exosome preparation from precipitation-based method whereas ultracentrifugation-based preparation appeared to be the purest. This data further supported the notion that exosome isolates from Invitrogen method likely carry microvesicle contamination, which could also be the reason for higher average size distribution. Thus to overcome this, a higher speed centrifugation step (15600 × g for 30 min.) should be incorporated to remove microvesicles prior to precipitation in Invitrogen method. However, while it should be noted that the recommendations of the MISEV 2018 guidelines currently encourages to denote “exosomes” as “small extracellular vesicles” because clear markers for delineation between microvesicles and exosomes are not established^32^. We continue to use the term exosomes in our work as the kits and methods used here have been suggested to isolate “exosomes”.

For qualitative assessment for downstream applications, we first measured RNA quality. Reverse transcription followed by PCR amplification for two mRNA (*GAPDH* and *ACTB*) and two small non-coding RNAs (U6 and miR-21) demonstrated comparable RNA quality among all the preparations. However, our mass spectrometry analysis suggested that Invitrogen precipitation-based method has Polyethylene Glycol (PEG), which hindered in mass spectroscopic analysis of exosomal proteins. The negative impact of PEG in Mass spectrometry analysis is also supported by others finding^28,33^. Lastly, we have evaluated the effect of exosomes on tumor cells viability and surprisingly, the exosomes isolated from Invitrogen method showed marginal toxicity which might be the presence of chemical component(s) presents in the Invitrogen precipitation reagent and pooled/precipitated with exosomes.

In conclusion, our comparative study has indicated that commercially available kit may be a possible alternative for quick and efficient isolation of exosomes from the limited amount of samples. All the kits mentioned the removal of cell, cell debris and apoptotic bodies but not much thought was given to potential contamination of moderate sized vesicles towards the isolation of exosomes except MagCapture method. To eliminate the microvesicles contamination and additional centrifugation step is required during sample processing. Similarly, an additional washing step might be required to get rid of chemical impurities coming from the precipitant in Invitrogen method; however, it might compromise its time and yield efficiency. Therefore, every method has its own pros and cons and one should consider making modification or choose the method wisely depending upon their intended use of exosomes for downstream applications.

## Materials and Methods

### Reagents and antibodies

RPMI-1640 cell culture media, penicillin, and streptomycin were procured from GE healthcare (Memphis, TN, USA), exosomes-depleted FBS-HI was from System Bio (Palo Alto, CA USA), Total Exosomes Isolation kit for culture media and total exosome RNA and protein isolation kit were from Invitrogen (Carlsbad, CA,USA), PureExo isolation kit was from 101Bio (Mountain View, CA, USA), MagCapture exosomes isolation kit was from Wako Life Sciences (Richmond, VA,USA), and qEV size-exclusion column was from iZON sciences (Cambridge, MA USA). The protein DC assay kit was procured from Bio-Rad (Hercules, CA, USA), and primary antibodies against CD9 (Ab2215) and ARF6 (Ab77581) were from Abcam (Cambridge, MA, USA). HRP-conjugated mouse- (SC-2005) and rabbit- (SC-2357) secondary antibodies were from Santa Cruz Biotechnology (Dallas, TX, USA). High fidelity RNA-cDNA kit was from Applied Biosystems (Foster City, CA, USA) and SYBR Green Real-Time PCR master mix was purchased from Applied Biosystems (Carlsbad, CA, USA). Western blotting SuperSignal WestFemto Maximum Sensitivity Substrate kit and protein concentrator centrifugal tube (MWCO, 3 kDa) were from Thermo Scientific (Rockford, IL USA), Immobilon-P PVDF membrane was from Millipore (Billerica MA, USA).

### Cell culture and exosome isolation

MiaPaCa cells were procured from ATCC (Manassas, VA, USA) and maintained in RPMI media supplemented with 5% FBS, 1% penicillin/streptomycin and grown in 5% CO_2_ at 37 °C. At 70% confluency in 100 mm culture dishes, cells were washed with PBS and RPMI media supplemented with exosomes-depleted FBS (5%) was replaced. After 48 h, culture supernatant was collected and centrifuged at 300 × g for 10 min to remove cellular debris. Subsequently, we made 5 ml aliquots for exosome isolation using different methods. Exosome isolation using classical ultracentrifugation method was done as described earlier by us^7^. For other commercial kits, we essentially followed the procedures suggested by their respective suppliers. However, we included one extra step in qEVsingle size-exclusion (iZON) chromatography method, since the column could not accommodate 5.0 ml volume. Therefore, we concentrated the supernatant to ~600 μl by centrifugation at 3000 × g for about 30 min using protein concentrator (MWCO 3 kDa).

### Size distribution, poly-dispersion index and zeta potential measurements

Size distribution and polydispersity index of isolated exosomes were measured by Dynamic Light Scattering (DLS) analysis on DelsaMax Pro (Beckman, CA, USA). In addition, we also recorded data for Phase Analysis Light Scatter (PALS) to determine zeta potential in water.

### Protein-based exosomes quantification and immunoblot analysis

Protein-based quantitation of isolated exosomes was done using the protein DC assay kit as described earlier by us^7,34^. Subsequently, equal amount of exosomes (25 μg) collected from different isolation methods was denatured using 6X denaturation buffer at 95 °C for 10 min and then resolved on 12% SDS-Polyacrylamide gel by electrophoresis. Resolved proteins were transferred onto Immobilon-P PVDF membrane and then blocked by incubating in 5% skimmed milk to minimize non-specific binding of antibodies. Blocked blots were submerged with primary antibodies for CD9 and ARF-6 overnight, and subsequently washed three times (10 min each) with 1X Tris buffer saline with 0.1% Tween-20 (TBST) buffer followed by incubation with HRP-conjugated secondary anti-mouse (for CD9) and anti-rabbit (for ARF-6) antibodies. Unbound antibodies were removed by washing with 1X TBST buffer (3 × 10 min), and signal recorded using WestFemto maximum sensitivity substrate kit under Bio-Rad ChemiDoc Imager (Hercules, CA, USA).

### RNA isolation, cDNA synthesis and quantitative real-time PCR (qRT-PCR) analysis

Total RNA was isolated using total exosome RNA isolation kit as per the manufacturer’s instructions and quantified using Nanodrop-1000 (Thermo Scientific, Waltham, MA, USA). The 40 ng RNA was used for cDNA synthesis using high capacity RNA-to-cDNA synthesis kit, where specific reverse transcription (RT) primers were used for U6 and miR-21, while random RT primers were used for cDNA synthesis for β-actin and GAPDH. 5 μl of cDNA was used as a template for PCR without dilution using CFX96 touch real-time PCR detection system (Bio-Rad, Hercules, CA, USA) in a total 20 μl reaction volume that included 10 μl of SYBR green qPCR master mix (2X) containing specific forward and reverse primers sets listed in Table 1. The thermal cycling conditions were as follows: cycle 1 at 95 °C for 10 min, and cycle 2 × 40): 95 °C for 10 s and 56 °C/60 °C for 45 s followed by melting curve detection. The detection of the fluorescence signal was represented in the form of cycle threshold (Ct).

### Qualitative Mass spectroscopic analysis

Exosome isolated from different methods were used to extract total proteins using 8 M urea treatment and sonication. Briefly, 30 µl of exosomes was dried using Speed-vac and suspended in 20 µl 8 M Urea followed by 10 minutes of sonication and incubated at −80 °C for 30 min to aid exosome lysis. Samples were further diluted to 4 times adding 60 µl of 50 mM Ammonium biocarbonate (ABC) and 10 mM Tris(2-carboxyethyl)phosphine (TCEP) to bring final urea concentration to 2 M. Overnight digestion was done 37 °C with 1.5 µl sequencing grade trypsin. Each sample subjected to a 1 h mass spectrometry analysis on Thermo QExactive Plus mass spectrometer for qualitative analysis to check the downstream application of exosomes for mass spectroscopy.

### Cell Proliferation Assay

To check the effect of isolated exosomes on cell proliferation, MiaPaCa Cells (5 × 10^3^ per well) were seeded in 96-well plate in the regular media. After attachment cells were washed and fresh media was replaced supplemented with exosomes-depleted FBS (5%) containing 10 µg/ml exosomes suspension and incubated for 72 h at regular culture conditions. After 72 h, proliferation assay was performed using WST-1 assay following manufacturer’s instructions.

### Statistical Analysis

All experiments were done with three independent biological replicates and data expressed as mean ± SD. Statistically significant differences were identified by one-way ANOVA using Tuckey’s multiple comparisons test on Prism, (GraphPad, La Jolla, CA). The p-value < 0.05 was considered as significant and individual values are presented in the respective graphs.

## Supplementary information

Supplementary Data

## Data Availability

We confirm that all the data in this manuscript is original, stored with us and is available for sharing upon a reasonable request.
