# Supplementary material for: Comparative analysis of exosome isolation methods using culture supernatant for optimum yield, purity and downstream applications
Source: Sci Rep. 2019 Mar 29;9:5335. doi: 10.1038/s41598-019-41800-2 (PMC6441044; doi:10.1038/s41598-019-41800-2)
Supplement: Supplementary file 1 — Supplementary Data [file 41598_2019_41800_MOESM1_ESM.pdf]

Comparative analysis of exosome isolation methods for optimum yield, purity and downstream applications

Girijesh Kumar Patel, Mohammad Aslam Khan, Haseeb Zubair, Sanjeev Kumar Srivastava, Moh'd Khushman, Seema Singh, Ajay Pratap Singh

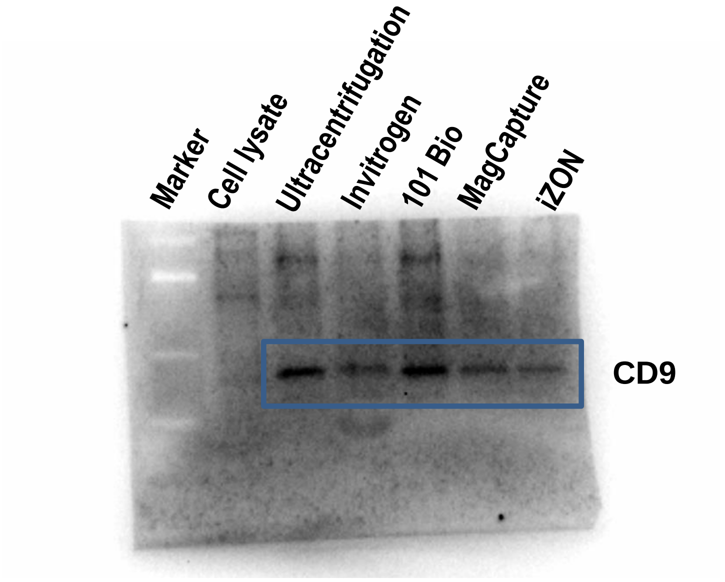

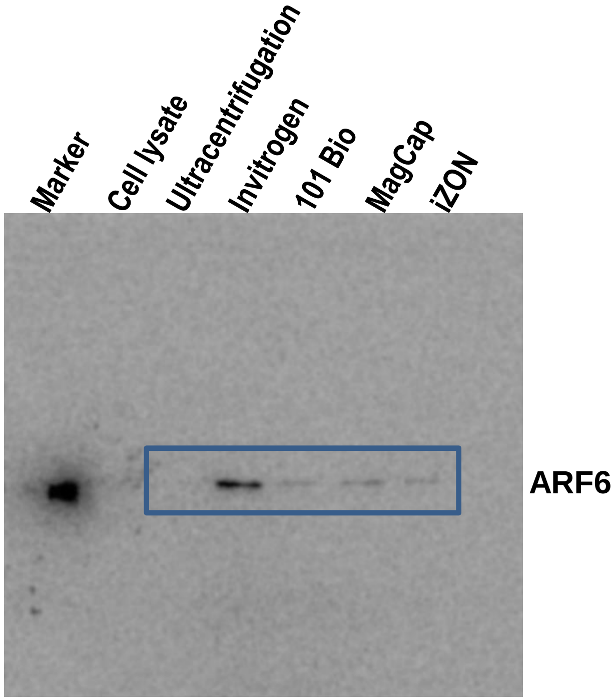

Supplementary Information: The full unedited gel blots used in the **Figure 4.** The cropped Bands (in the blue boxes) are reported as Figure 4.
